# Preliminary molecular detection of influenza RNA in synanthropic cockroaches from shiraz, Iran

**DOI:** 10.1016/j.parepi.2026.e00498

**Published:** 2026-03-26

**Authors:** Mohsen Kalantari, Mozaffar Vahedi, Marzieh Jamalidoust, Maryam Motevasel, Amin Hosseinpour

**Affiliations:** a Research Center for Health Sciences, Institute of Health, Department of Biology and Control of Disease Vectors, School of Health, Shiraz University of Medical Sciences, Shiraz, Iran; b Clinical Microbiology Research Center, Shiraz University of Medical Sciences, Shiraz, Iran; c Diagnostic Laboratory Sciences and Technology Research Center, Faculty of Paramedical Sciences, Shiraz University of Medical Sciences, Shiraz, IR, Iran

**Keywords:** Cockroaches, Influenza, **Mechanical carriage**, Real-time PCR, Shiraz, Iran

## Abstract

Cockroaches are recognized as significant mechanical vectors for a wide spectrum of pathogens, posing a considerable public health risk. Their habitation in unsanitary environments and promiscuous feeding habits allow them to acquire and disseminate bacterial agents, viruses—including poliovirus and influenza—and protozoan parasites such as *Microsporidia* and *Giardia*, as well as the eggs of parasitic worms. This study investigates the potential of cockroaches to mechanically carry influenza viruses, a subject that remains underexplored despite the significant global burden of influenza. During the seasonal peak of influenza activity, a total of 322 cockroaches were collected from various high-risk locations in Shiraz, Iran, including hospital premises, university dormitories, and academic faculties. The sampling targeted two predominant species, *Blattella germanica* and *Periplaneta americana*, captured from diverse microhabitats such as kitchens, rooms, and sewers. Using a highly sensitive real-time PCR methodology, which targeted the conserved matrix genes of influenza A and B viruses, both external body surface washes and internal digestive tract samples were analyzed. The results confirmed the presence of influenza A virus RNA in four external surface samples of *Blattella germanica* and in two internal digestive tract samples of *Periplaneta americana*. Notably, the majority of positive samples (5 out of 6) were for influenza type A, with one sample positive for influenza type B. The overall detection rate was low (1.86%, 6/322), these findings demonstrate that cockroaches in urban environments can indeed harbor **influenza virus RNA**, either externally on their bodies or internally within their digestive systems. This molecular detection highlights the presence of viral RNA, suggesting possible mechanical carriage. However, the detection of RNA alone does not confirm the presence of viable, infectious virus, which is a key limitation of this study. Given their intimate association with human dwellings and food sources, this molecular evidence indicates potential but unconfirmed route for mechanical carriage that warrants further investigation. Crucially, our study design cannot assess transmission risk. Consequently, the findings underscore the necessity for additional comprehensive studies **to assess viral viability and to elucidate the potential, yet unproven, role** of cockroaches in the epidemiology **of influenza.**

## Highlights

• First molecular detection of Influenza virus **RNA** in Iranian cockroaches.
• **Viral RNA** found on cockroach exteriors and inside their digestive tracts.
• Both Influenza A and B **RNAs** were detected.
• Low detection rate (1.86%) of influenza virus RNA in cockroaches suggests mechanical carriage may be sporadic
• It needs more studies to confirm the presence of viable and infectious of influenza virus in cockroaches.

## 1 Introduction

Influenza, also known as the flu, is a contagious disease caused by a type of virus from the Orthomyxoviridae family. This virus can cause disease in birds and mammals, primarily affecting the respiratory system. Influenza is one of the most common respiratory infections, affecting many people and, consequently, mortality (Yanni et al., 2010). The influenza virus has three types: A, B, and C, with type A being responsible for pandemics and multiple worldwide outbreaks in recent years (Rejali et al., 2011). The genetic changes in this virus occur through a process of shift and drift, which makes it different from any other virus and requires the annual production of vaccines against it (Yalda et al., 2006). Influenza has manifested as several pandemics throughout history. The first pandemic of the new influenza A (H1N1) occurred in early March 2009 in Mexico, causing its spread worldwide and significant human and financial damages (Michaelis et al., 2009). This pandemic strain arose from a major genetic re-assortment (antigenic shift) involving avian, human, and swine influenza viruses, underscoring the critical role of cross-species transmission and host-virus interactions in the emergence of novel influenza A variant. According to a report from the World Health Organization in November 2016, between 5 and 3 million people worldwide were infected with this disease, with an estimated 500,000 to 250,000 deaths as a result (Hatami et al., 2009).

Influenza is an acute respiratory infection that causes sudden headaches, muscle pain, fever, and severe weakness and fatigue. Type A affects humans and other mammals and birds, type B primarily affects humans, and type C is typically seen in humans (Ungchusak et al., 2012). The International Committee on Taxonomy of Viruses (ICTV) has classified influenza viruses into distinct genera and species: *Alphainfluenzavirus influenzae* (Influenza A virus), *Betainfluenzavirus influenzae* (Influenza B virus), *Gammainfluenzavirus influenzae* (Influenza C virus), and *Deltainfluenzavirus influenzae* (Influenza D virus), with Influenza D primarily affecting cattle**.** Usually, the disease period lasts about 3 to 4 days. Influenza is a short-term respiratory infection that can be severe in all individuals, especially adults. It can be life-threatening for the elderly, children, and those with underlying cardiovascular or respiratory diseases. If left uncontrolled, it can lead to death (Ungchusak et al., 2012).

Individuals with underlying health conditions such as heart, lung, or kidney disease, diabetes, asthma, as well as pregnant women and the elderly, face an increased likelihood of contracting influenza. Children are also more susceptible to this disease due to their developing immune systems (Alizadeh et al., 2010). Transmission primarily occurs via respiratory droplets and aerosols. However, fomites—contaminated inanimate objects—play a significant supplementary role in virus spread, as influenza viruses can persist on surfaces for hours to days. This underscores the potential importance of mechanical vectors, which can act as mobile fomites (Van et al., 2010). Transmission of avian influenza A virus to humans may also occur through contact with intermediate hosts such as domestic poultry or swine. Furthermore, indirect transmission via contaminated surfaces (fomites) or, in the case of some zoonotic strains, through exposure to contaminated environments or undercooked animal products, represents another potential route.

The incubation period for influenza, from viral entry to the onset of symptoms, is typically ∼24–48 h (approximately 1–4 days) for seasonal human influenza A and B viruses (Centers for Disease Control and Prevention, 2013). This period can vary slightly; for Influenza A, it is generally 1–4 days, while for Influenza B, it is often similar, though some reports indicate a potentially shorter average duration (Uyeki et al., 2022). While three primary types exist (A, B, and C), these viruses are prone to mutation, leading to the emergence of different strains. Influenza A virus, in particular, has a broad host range and is classified into subtypes based on its surface glycoproteins, hemagglutinin (H) and neuraminidase (N). Certain zoonotic subtypes, such as avian influenza A(H5) and A(H7), are classified as highly pathogenic avian influenza (HPAI) viruses. These HPAI strains can cause severe respiratory illness in humans, with a high case-fatality rate, often presenting with rapid progression to pneumonia, acute respiratory distress syndrome (ARDS), and multi-organ failure (Uyeki et al., 2022; World Health Organization, 2016). Seasonal epidemics occur almost every winter, with varying severity (Thompson et al., 2009; Nair et al., 2011). The disease can affect individuals of any age and gender, and its presentation ranges from mild to severe. Common symptoms include fever, chills, headache, myalgia (including back pain), fatigue, cough (which may be productive), sore throat, a raspy voice, and coryza. Gastrointestinal symptoms such as nausea and vomiting are more frequent in children than in adults. While most symptoms usually resolve within a week, the cough may persist for two weeks or longer. The combination of systemic symptoms like significant myalgia and lethargy, which can be severe enough to cause prostration, helps distinguish influenza from other common respiratory infections (Thompson et al., 2009; Nair et al., 2011).

In Mexico, the starting point of the pandemic, almost 200,000 positive samples of influenza A with 2000 deaths were reported (Borja-Aburto et al., 2012; Dávila-Torres et al., 2015; Ríos-Silva et al., 2022). High mortality rates were also observed in other countries, including India (11%), Guatemala (2.7%), and Brazil (1.8%) (Ferrante et al., 2011). Iran was not spared from this pandemic (Rejali et al., 2011) and the first case of influenza type A (H1N1) was confirmed on June 26, 2009 (Ferrante et al., 2011; Afzali et al., 2011). A total of 3672 cases of influenza A were reported within 4 months, with a mortality rate of less than 3% (140 people) (Yanni et al., 2010; Rejali et al., 2011; Yalda et al., 2006; Ungchusak et al., 2012; Hatami et al., 2009). The most common clinical symptoms of influenza at the beginning of the 2009 pandemic were fever, cough, sore throat, fatigue, and headache. Diarrhea, nausea, and vomiting are relatively common in seasonal influenza but were observed more frequently in the new influenza strain (Picone et al., 2009).

The main route of virus transmission in crowded environments is airborne (respiratory droplets). The virus can be detected in the respiratory secretions of an infected person 24 h before the onset of symptoms (Wang et al., 2021), and it can be transmitted for more than 5 days after the onset of the disease (Javaheri et al., 2012).

Preventive measures such as education, increasing people's awareness, and developing preventive skills for personal protection against this disease are some of the most important and necessary strategies for influenza disease prevention and control programs. Choosing a health education model is the first step in the planning process of any health education program, and the right model will keep the program on the right track and in the right direction. One of the pedagogical models in health education is the Health Belief Model (Guvenc et al., 2011). This model emphasizes how a person's perception creates motivation and movement and triggers behavior in them. In general, this model focuses on changing beliefs, leading to a change in behavior (Guvenc et al., 2011). According to this model, to take preventive action, people must first come to terms with the problem, i.e., they must sense the risk of flu (perceived sensitivity), then understand the depth of this risk and the severity of the various complications in their physical, psychological, social and economic dimensions (perceived severity), as well as with the positive symptoms that come from the environment or their internal environment (practice guide), believe that the flu prevention program is useful and applicable (perceived benefits), and find that the factors preventing the action are less costly than its benefits (perceived barriers), so they end up taking preventive measures against influenza disease.

Cockroaches are insects that feed on rotting food and waste and regurgitate some of their undigested food. The most important species with health and medical significance include the American cockroach, the German cockroach, the Oriental cockroach and the Egyptian cockroach (Martínez-Girón et al., 2017; Santa Cruz and Sfara, 2018; McPherson et al., 2021).

Beyond direct person-to-person transmission via respiratory droplets, influenza viruses can be spread indirectly through contaminated surfaces and mechanical hosts. Documented evidence shows that influenza A and B viruses can persist on various environmental surfaces, with their stability influenced by factors such as temperature, humidity, and the nature of the material. Furthermore, environmental mechanisms such as air currents and the resuspension of settled dust may contribute to the dispersal and persistence of viral particles. Of particular relevance are synanthropic insects, such as flies, which have been identified as potential mechanical hosts. These insects can acquire and harbor viable virus particles on their exoskeletons or within their digestive tracts after contact with contaminated substrates, thereby potentially contributing to the maintenance and transmission of influenza viruses in human environments (Chamavit et al., 2011; Oyeyemi et al., 2016; Salehzadeh et al., 2007; Nayduch et al., 2026).

Numerous studies have documented the isolation or molecular detection of various viral agents from cockroaches, including enteroviruses, rotaviruses, and coronaviruses, reinforcing their capacity as mechanical vectors. Mechanical carriage involves the passive transport of pathogens on the body surface (exoskeleton) or within the digestive tract without viral replication in the vector, distinct from biological transmission where the pathogen replicates within the vector. In contrast to the data on enteric and other viruses, evidence regarding the association of cockroaches with influenza viruses remains notably scarce. This study therefore aimed to conduct a molecular survey to investigate the presence of influenza A and B virus RNA on and within synanthropic cockroaches collected from urban environments in Shiraz, Iran. The goal was to provide initial evidence to assess whether cockroaches could be implicated in the mechanical carriage of this major respiratory pathogen.

**This study aimed to investigate the presence of influenza A and B virus RNA on the external surface and within the digestive tract of cockroaches collected from high-risk urban environments in Shiraz, Iran. Given the synanthropic nature of cockroaches, their potential to mechanically carry respiratory virus RNA remains a public health consideration. We hypothesized that cockroaches, due to their habitat and feeding behavior, could harbor influenza virus RNA on their external surfaces or within their digestive tracts. Using real-time PCR, we examined samples from** *Blattella germanica* **and** *Periplaneta americana* **collected from hospital premises, university dormitories, and academic faculties during the seasonal influenza peak.**

## 2 Material and methods

### 2.1 Study design and sampling strategy

**Fig. 1:**
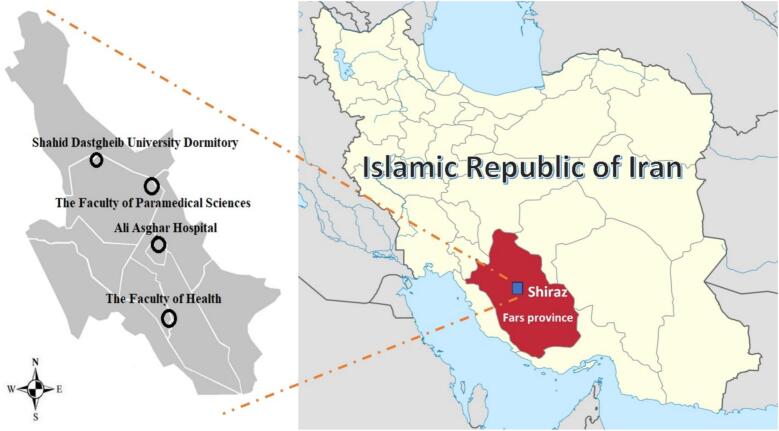
Map of studied areas in Shiraz, southern Iran.

This descriptive cross-sectional study was designed to systematically assess the presence of influenza virus on synanthropic cockroaches in Shiraz, Iran. Sampling was conducted during the seasonal peak of influenza activity in the region, specifically from November 2022 to January 2023**.** During this period, the average ambient temperature in Shiraz ranges from 5 °C to 15 °C, with relatively low humidity. The selected sites represent environments with varying degrees of human activity and hygiene standards, from hospital wards and kitchens (with regular cleaning) to sewer systems and dormitory basements (with potentially lower hygienic control). Sampling sites were purposively selected based on the following criteria: 1) high human traffic and density, 2) locations with a higher potential for influenza virus circulation (e.g., hospitals), and 3) environments with known or reported cockroach infestations. Four primary locations meeting these criteria were chosen: the premises of Ali Asghar Hospital, Shahid Dastgheyb University Dormitory, the Faculty of Paramedical Sciences, and the Faculty of Health. Within each location, sampling targeted three predefined microhabitats where cockroaches are commonly found: kitchens/dining areas, residential/patient rooms, and sewer systems or dark, moist basements (Fig. 1).

### 2.2 Collection protocol

A standardized collection protocol was applied across all sites. For *B. germanica* in indoor areas, non-toxic sticky traps (Blackhole, Kness Mfg., USA) baited with a standardized bread and beer attractant were deployed overnight in kitchens and rooms (10 traps per microhabitat per location). For *P. americana* in sewers, a standardized manual collection method was employed following the application of a mild irritant spray (pyrethroid-based) to facilitate capture. Traps were placed in fixed, concealed positions within each microhabitat and left for 72 h. Sewer specimens were collected using a modified technique where irritant sprays (pyrethroid-based) were briefly applied at sewer entrances to drive cockroaches out, followed by immediate manual collection into sterile containers. All collected specimens were immediately placed in individual sterile 50 ml conical tubes, transported on ice to the laboratory, and identified to species level using taxonomic keys (Cochran, D.G. and World Health Organization, 1999).

### 2.3 Sample collection and processing

#### 2.3.1 Surface decontamination and external wash

Upon arrival at the laboratory, cockroaches designated for external body surface analysis were subjected to a two-step decontamination procedure to remove potential environmental contaminants prior to virus elution. First, each specimen was briefly immersed in 70% ethanol for 10 s and air-dried under a laminar flow hood. Subsequently, the external wash was performed by vortexing each cockroach for 2 min in a 15 ml tube containing 5 ml of sterile phosphate-buffered saline (PBS) supplemented with 0.1% Tween 20. The wash solution was then centrifuged at 3000 ×*g* for 10 min, and the supernatant was aliquoted for RNA extraction. A sterile PBS-Tween solution processed identically but without a cockroach served as an environmental/field negative control for every batch of 10 samples.

#### 2.3.2 Digestive tract dissection

For internal samples, a separate set of cockroaches was surface-sterilized by immersion in 70% ethanol for 60 s, followed by three rinses in sterile distilled water. Dissections were performed under sterile conditions using autoclaved instruments on a fresh, disinfected surface for each specimen. The entire digestive tract, from the crop to the hindgut, was aseptically removed, homogenized in 1 ml of sterile PBS using a sterile pestle, and clarified by centrifugation. The supernatant was used for RNA extraction.

#### 2.3.3 Sample size calculation

The target sample size was calculated using the formula for estimating a population proportion: n = Z2∗P(1 − P)/d2n = Z2∗P(1 − P)/d2, where **Z is the *Z*-value for the confidence level (1.96 for 95% CI), P is the estimated prevalence, and d is the desired precision.** Based on a previous study in Iran that reported a high prevalence (approximately 70%) of various microbial agents on cockroaches (Salehzadeh et al., 2007), and considering the novel nature of investigating influenza, we used a conservative estimated prevalence (P) of 0.5 (50%) to maximize the required sample size. With a 95% confidence level (Z = 1.96) and a desired precision (d) of 0.05 (5%), the calculation yielded a minimum sample size of 385. However, due to logistical constraints in systematic collection, we collected 322 cockroaches, which provides a precision of approximately ±5.4% for a 50% prevalence estimate, which we deemed acceptable for this preliminary survey. Efforts were made to collect a comparable number of the two predominant species, *Blattella germanica* (commonly found indoors) and *Periplaneta americana* (common in sewers and basements), from their respective preferred microhabitats. This number was proportionally allocated across the four main locations.

### 2.4 Molecular detection of influenza virus

#### 2.4.1 RNA extraction and reverse transcription

Total RNA was extracted from 200 μl of each supernatant (external wash or digestive tract homogenate) using the QIAamp Viral RNA Mini Kit (Qiagen, Germany) according to the manufacturer's instructions, including the addition of carrier RNA. Extractions were performed in a dedicated pre-PCR clean room equipped with UV light and separate from all post-PCR areas. For every extraction batch, one negative control (sterile nuclease-free water) was processed alongside the samples to monitor for cross-contamination during extraction. RNA was eluted in 60 μl of AVE buffer and stored at −80 °C. Reverse transcription to cDNA was performed using the PrimeScript RT reagent Kit (Takara Bio, Japan) with random hexamers.

#### 2.4.2 Real-time PCR assay

The real-time PCR protocol targeting the matrix genes of influenza A and B viruses was performed as previously described (Boivin et al., 2004), utilizing only the influenza A and influenza B components of the multiplex assay. The primer and probe sequences, along with their final concentrations and expected amplicon sizes, are detailed in the table (Table 1). All PCR setup was conducted in the pre-PCR clean room using aerosol-barrier tips. Each 25 μL reaction mixture contained 12.5 μL of 2× QuantiTect Probe RT-PCR Master Mix (Qiagen, Germany), 0.5 μL of QuantiTect RT Mix, the specified concentrations of primers and probes, 5 μL of extracted RNA, and nuclease-free water to volume. Each reaction run included: (1) the test samples, (2) a negative extraction control (nuclease-free water processed through the RNA extraction kit), (3) a no-template control (NTC) containing nuclease-free water instead of RNA, and (4) a positive control consisting of quantified inactivated influenza A (H1N1) and influenza B (Victoria lineage) RNA (Vircell, Spain). Thermal cycling and fluorescence detection were carried out on a LightCycler® 480 II instrument (Roche Diagnostics, Switzerland) in a separate post-PCR room with the following cycling conditions: reverse transcription at 50 °C for 30 min; initial PCR activation at 95 °C for 15 min; followed by 45 cycles of denaturation at 94 °C for 15 s and annealing/extension at 55 °C for 1 min (with single fluorescence acquisition). A sample was considered positive for influenza virus RNA if it produced a characteristic exponential amplification curve with a cycle threshold (Ct) value <40. Furthermore, all corresponding negative controls (environmental field blank, extraction control, and NTC) from the same run were required to show no amplification (Ct = 0 or undetermined).

**Table 1:** Oligonucleotide primers and TaqMan probes used for real-time RT-PCR detection of influenza viruses.

Target Virus	Primer/Probe Name	Sequence (5′ → 3′)	Final Concentration (nM)	Amplicon Size (bp)	Reference
Influenza A	InfA-F	GAC CRA TCC TGT CAC CTC TGA C	900	104	(Boivin et al., 2004)
	InfA-R	AGG GCA TTY TGG ACA AAK CGT CTA	900		
	InfA-P	**FAM**-TGC AGT CCT CGC TCA CTG GGC ACG-**BHQ1**	200		
Influenza B	InfB-F	TCC TCA AYT CAC TCT TCG AGC G	900	102	(Boivin et al., 2004)
	InfB-R	CGG TGC TCT TGA CCA AAT TGG	900		
	InfB-P	**Cy5**-CCA ATT CGA GCA GCT GAA ACT GCG GT-**BHQ2**	200		

^⁎^ Abbreviations: FAM, 6-carboxyfluorescein; Cy5, cyanine5; BHQ, Black Hole Quencher.

## 3 Results

A total of 322 cockroaches, comprising *Blattella germanica* and *Periplaneta americana*, were tested for viral presence using external body washes and internal digestive tract samples. The collected specimens included 258 adults, 48 nymphs, and 16 ootheca. The sample distribution across collection sites was as follows: 81 from Shahid Dastgheyb Dormitory (65 adults, 12 nymphs, 4 ootheca), 80 from Ali Asghar Hospital (64 adults, 9 nymphs, 7 ootheca), 81 from the Faculty of Paramedical Sciences (72 adults, 5 nymphs, 3 ootheca), and 80 from the Faculty of Health (54 adults, 17 nymphs, 9 ootheca) (Table 2, Table 3).

**Table 2:** The number of cockroach samples collected at different locations in Shiraz.

Sampling Locations	Species	Sampling Places			Total Number
		Kitchen/ Dining Room	Rooms	Sewer	
Dormitory of Shahid Dastgheyb	*P. americana*	0	0	40	40
	*B. germanica*	36	5	0	41
Ali Asghar Hospital	*P. americana*	0	0	40	40
	*B. germanica*	35	5	0	40
Paramedical faculty	*P. americana*	0	6	34	40
	*B. germanica*	34	7	0	41
Faculty of Health	*P. americana*	0	3	37	40
	*B. germanica*	36	4	0	40
Total number		141	30	151	322

**Table 3:** The results of Real-Time PCR tests, the number of samples and sampling location, and samples infected with influenza in Shiraz.

Sampling Locations	Species	Total No. of tested	Number of positive Cockroaches in Real-Time-PCR assay			
			External body surfaces		Internal tissue samples	
			Type B influenza	Type A influenza	Type B influenza	Type A influenza
Dormitory of Shahid Dastgheyb	*P. americana*	40	0	0	0	1
	*B. germanica*	41	0	2	0	0
Ali Asghar Hospital	*P. americana*	40	0	0	0	0
	*B. germanica*	40	0	0	0	0
Faculty of Paramedicine	*P. americana*	40	0	0	0	0
	*B. germanica*	41	0	1	0	0
Faculty of Health	*P. americana*	40	0	0	1	0
	*B. germanica*	40	0	1	0	0
Total number		322	0	4	1	1

All negative controls (environmental field blanks, extraction controls, and no-template PCR controls) remained negative throughout the study, confirming the absence of cross-contamination during sample processing and analysis. It is important to emphasize that a positive PCR signal indicates the presence of viral RNA but does not distinguish between viable virus and non-infectious genetic material. Furthermore, the amplicons were not sequenced, which precludes definitive confirmation of target identity and cannot entirely exclude the possibility of non-specific amplification, though the use of TaqMan probes increases assay specificity.

### 3.1 Statistical analysis of detection rate

The overall prevalence of influenza A and B virus RNA in the collected cockroaches was 1.86% (1.55% for Flu A (5/322), 0.31% for Flu B (1/322). The 95% confidence interval (CI) for this proportion, calculated using the efficient-score method (Wilson score interval), is 0.69% to 4.00%. This low detection rate indicates that while molecular evidence of viral RNA carriage was confirmed, it was an infrequent finding in this sample population.

**Fig. 2:**
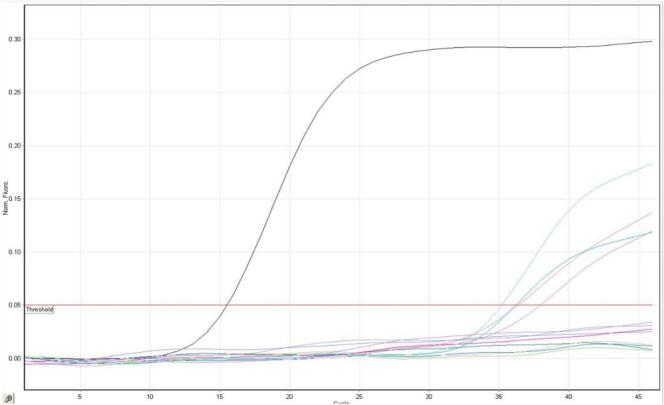
Real Time-PCR diagram of influenza type A cases.

**Fig. 3:**
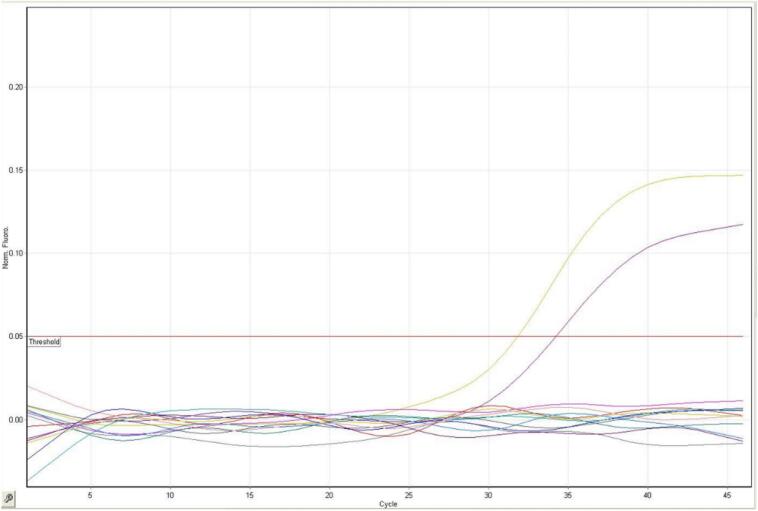
Real Time-PCR diagram of influenza type B cases.

In the samples prepared from the body surface of the German cockroach, 4 positive samples of influenza type A RNA were detected in the Dastgheyb dormitory (one case of Ootheca and one case of Nymph), in the Faculty of Health (1 case of adult) and the Faculty of Paramedical Sciences (1 case of Ootheca). In the sample prepared by the American cockroach, one positive sample of influenza A RNA was observed in the sample collected from the Dastgheyb dormitory and one positive sample of influenza type B RNA was observed in the sample collected from the Faculty of Health (Fig. 2, Fig. 3).

The detection of RNA within the digestive tract represents ingestion of virus-contaminated environmental material and does not indicate active infection or internal persistence of the virus within the cockroach.

## 4 Discussion

This study presents the molecular detection of influenza A virus RNA on the external surface and within the digestive tract of field-collected cockroaches (*Blattella germanica* and *Periplaneta americana*) in Iran. While the detection rate was low (6/322, ∼1.86%), it confirms that cockroaches can harbor influenza virus genetic material in urban settings. This finding aligns with their known behavior as mechanical carriers of diverse pathogens. The methodological approach included stringent controls, including surface decontamination, separate pre- and post-PCR facilities, and multiple negative controls, which lend confidence to the specificity of the molecular detections reported.

However, a key limitation is that the detected nucleic acid was not sequenced to confirm its identity as influenza virus RNA, nor were attempts made to culture viable virus from positive samples. Therefore, while the results suggest mechanical carriage of viral material, they do not confirm the presence of infectious virus or rule out the possibility of PCR artifacts with absolute certainty.

The low prevalence (1.86%, 95% CI: 0.69–4.00%) warrants careful epidemiological interpretation. This rate is substantially lower than detection rates reported for more environmentally stable bacterial or parasitic agents on cockroaches from similar settings, which often exceed 50% (Salehzadeh et al., 2007). The 1.86% detection rate likely reflects the confluence of viral, environmental, and methodological factors. Firstly, regarding environmental contamination and seasonality, while sampling was conducted during the regional seasonal peak (November–January), the actual prevalence of influenza RNA in the environment is not uniform. Human viral shedding and subsequent environmental contamination are transient events. Cockroaches may only intermittently encounter freshly contaminated surfaces or secretions, especially in microhabitats like sewers or basements that are removed from direct human respiratory droplet deposition. Secondly, the stability of influenza viruses on surfaces is relatively short (hours to a few days) and highly sensitive to temperature, humidity, and sunlight (Kampf et al., 2020). The conditions in many cockroach habitats (e.g., cool, dark sewers) might prolong survival somewhat, but the overall temporal window for acquisition of viral RNA is narrow compared to hardy bacterial spores or protozoan cysts. Thirdly, our methodological decontamination step, while essential for specificity, may have reduced the recovery of superficially adherent viral material. Finally, this low rate may indeed indicate that acquisition of influenza virus RNA by cockroaches is a sporadic event under typical urban conditions. However, even a low prevalence confirms the **detection** of **viral RNA**. In high-risk, high-density environments such as hospital wards during an intense outbreak, where environmental contamination is frequent and concentrated, the likelihood of cockroach exposure and potential RNA acquisition could be higher. Thus, the 1.86% rate should be viewed as a baseline of RNA detection under general seasonal conditions, not as an absolute measure of risk, which would be context-dependent.

Viruses can be spread directly via infected secretions or large aerosol droplets (Gomes, 2020; Reed, 1984). In addition, infected individuals can contaminate surfaces they encounter, including many household items and appliances (Gomes, 2020). Early studies show that viruses can survive on surfaces for hours to days, with survival time dependent on various conditions, such as the type of surface, ambient temperature, and humidity (Kampf et al., 2020). The detection of viral RNA on the cockroach exterior is consistent with their movement over contaminated surfaces, acting as mobile fomites. The finding of RNA within the digestive tract suggests ingestion of contaminated material, such as sputum or other secretions.

This internal detection reflects passive ingestion of environmental contaminants rather than active infection or any biological process supporting the virus. These findings represent molecular evidence of environmental contamination associated with cockroaches. They align with the broader concept of insects acting as passive carriers of pathogens genetic material, a phenomenon documented for other viruses and insect species (Salehzadeh et al., 2007). However, this study does not constitute evidence of mechanical transmission, which would require demonstration of viable virus transfer. Any public health implications regarding transmission are speculative and not supported by our data.

### 4.1 Distinction between biological vector and mechanical host

It is crucial to distinguish the potential role identified here from that of a biological vector. In medical entomology, a true vector, typically a hematophagous arthropod like a mosquito or tick, possesses both *competence* (the biological ability to support pathogen replication and development) and *vectorial capacity* (a composite measure of the efficiency of transmission within a specific ecological context). In contrast, a mechanical host or carrier passively acquires and transiently harbors pathogens on its body surface (exoskeleton) or within its digestive tract without any biological amplification or developmental cycle of the pathogen (Garcia, 2008). The synanthropic cockroaches in this study exemplify the latter. The detection of influenza virus RNA on their exteriors and in their guts is consistent with mechanical acquisition from contaminated environmental substrates—acting as mobile fomites—or through passive ingestion. Their biology does not support influenza virus replication; thus, they lack vector competence. Therefore, while our results provide molecular evidence for mechanical carriage, they do not suggest that cockroaches are biological vectors for influenza viruses. This distinction underscores that any potential public health risk would stem from their role in the mechanical translocation of viral particles, not from perpetuating a transmission cycle involving viral replication within the insect host.

Furthermore, while sampling was conducted during the influenza season, we did not quantitatively measure environmental parameters such as temperature and humidity at each microhabitat, nor did we formally assess the hygienic conditions. These factors are known to influence viral stability on surfaces and could affect the likelihood of cockroaches acquiring and carrying viable virus. Future studies should document these variables to better understand the environmental context of mechanical carriage.

### 4.2 One health implications of the findings

The detection of influenza A virus RNA in synanthropic cockroaches, as reported here, should be interpreted within a One Health framework, which recognizes the interconnected health of humans, animals, and their shared environments (Destoumieux-Garzón et al., 2018). Cockroaches thrive at the nexus of these domains: they inhabit human dwellings and sewers (environmental reservoir), can acquire pathogens from contaminated waste or surfaces, and have direct access to human food and living spaces. The presence of viral RNA on these insects serves as a bioindicator of environmental contamination with a human respiratory pathogen. While the direct transmission risk remains unquantified, this finding underscores a potential indirect pathway in the influenza transmission network, particularly in dense urban settings. It reinforces the concept that effective influenza surveillance and control may need to extend beyond clinical human cases and domestic animal populations to include monitoring of urban pest populations and environmental hygiene. Addressing such a potential route requires integrated strategies spanning public health education, urban pest management, and environmental sanitation—core tenets of a One Health approach to reducing zoonotic and environmental disease threats.

### 4.3 Biological and environmental considerations for potential virus carriage

The interpretation of positive samples necessitates an examination of the factors that could facilitate the maintenance and potential transmission of viable virus. Biologically, the waxy cuticle of the cockroach exoskeleton may provide transient protection to viral particles against desiccation. Furthermore, their foraging within cool, humid microhabitats—such as sewers and basements—aligns with environmental conditions known to prolong the survival of enveloped viruses like influenza on surfaces. Reported viability on fomites ranges from several hours up to 48 h, contingent upon temperature, humidity, and the presence of organic material. This window of persistence allows a plausible timeframe for cockroaches to acquire viable virus from recently contaminated surfaces. Their feeding behavior also leads to the passive internalization of environmental contaminants; although the gut environment is generally inhospitable, the detection of RNA signifies prior ingestion of contaminated matter, not infection (Kampf et al., 2020). Based on these observations, two indirect transmission scenarios can be proposed: cockroaches may mechanically transfer virions from contaminated surfaces in high-risk settings (e.g., hospital wards) to nearby food or utensils (external carriage), or they may deposit virus-laden feces or regurgitant onto surfaces following the ingestion of contaminated matter (internal carriage). Thus, cockroaches could act as mobile reservoirs, potentially amplifying and dispersing environmental contamination.

The key limitation of our study is the use of PCR, which precludes any conclusion regarding the viability or infectivity of the detected influenza virus RNA. The RNA could originate from inactivated virus particles. Therefore, our findings indicate mechanical carriage but not confirmed mechanical transmission. Furthermore, the sample size, while calculated, yielded a low frequency of detection, suggesting this may be a sporadic event. The study design did not allow for tracing the source of the virus to specific human cases.

However, a key and central limitation is that the detected nucleic acid was not sequenced to confirm its identity as influenza virus RNA, nor were attempts made to culture viable virus from positive samples. Therefore, while the results suggest mechanical carriage of viral genetic material, they do not confirm the presence of infectious virus, rule out PCR artifacts with absolute certainty, or provide evidence of transmission potential. The critical question of viral viability remains entirely unanswered by this molecular survey.

The decision not to sequence was primarily based on the very low viral load indicated by high Ct values (Ct > 35) in the positive samples, which often yields insufficient template for reliable Sanger sequencing. Additionally, as a preliminary survey, the study's primary aim was initial screening for presence/absence of viral RNA; strain identification and definitive confirmation via sequencing are recommended priorities for future, more extensive studies.

Future investigations should focus on: 1) Culturing attempts from PCR-positive samples to confirm the presence of infectious virus, 2) Experimental studies to assess the duration of viral persistence and potential for mechanical transfer by cockroaches under controlled conditions, and 3) Larger-scale epidemiological surveys during influenza outbreaks to correlate cockroach contamination with human cases. Such studies are essential to move from molecular detection to a substantive understanding of transmission risk.

## 5 Conclusion

This preliminary study provides the first molecular evidence of influenza A virus RNA associated with cockroaches in Iran. The detection of viral RNA on external surfaces and within the digestive tract provides molecular evidence of environmental contamination and ingestion, respectively. The detection of viral RNA on external surfaces and within the gut suggests environmental contamination and passive ingestion, respectively. However, the low prevalence rate (1.86%) indicates that such detections may be infrequent under the studied conditions. The central limitation is that our PCR-based method cannot confirm viral viability or infectiousness, and no such assessments were performed. Therefore, the detection of RNA does not constitute evidence of transmission risk. The critical question of whether cockroaches can carry and transmit viable influenza virus remains unanswered, as our PCR-based method cannot confirm viability nor, without sequencing, provide definitive proof of target origin. Our findings should not be interpreted as demonstrating a transmission pathway but rather as identifying **the presence of viral genetic material** in an environmental context that justifies further investigation under a One Health framework. Future studies must include viral culture and infectivity assays to move from molecular detection to a substantive understanding of any potential epidemiological role.

Cockroaches are among the most widespread insects in the world and are cosmopolitan. Due to their gregarious nature and homotropic preferences, cockroaches are usually found in or near human dwellings. They are also promiscuous feeders, transferring microbial pathogens from litter-contaminated breeding habitats to the human food that humans use as nourishment, which the human host can eat. Infection with the virus leads to severe gastrointestinal infections in some people, and the virus is detected in their stool. Given the documented ability of cockroaches to mechanically carry various pathogens, and the molecular detection of influenza virus RNA in cockroaches, it appears necessary to investigate additional studies in this field.

## CRediT authorship contribution statement

**Mohsen Kalantari:** Visualization, Validation, Supervision, Project administration, Methodology, Conceptualization, Writing – original draft. **Mozaffar Vahedi:** Validation, Investigation, Data curation, Writing – review & editing. **Marzieh Jamalidoust:** Resources, Investigation, Formal analysis, Writing – review & editing. **Maryam Motevasel:** Resources, Methodology, Writing – review & editing. **Amin Hosseinpour:** Software, Data curation, Writing – review & editing.

## Ethical issues

All subjects gave informed consent for inclusion before participating in the study. The study was conducted following the Declaration of Helsinki and the protocol was approved by the Ethics Committee of Shiraz University of Medical Sciences (IR.SUMS.SCHEANUT.REC.1402.080) and the Research project code is 28507.

## Declaration of competing interest

The authors declare that they have no conflict of interest.
